# Should infectious disease modelling research be subject to ethics review?

**DOI:** 10.1186/s13010-023-00138-4

**Published:** 2023-08-04

**Authors:** Ben Green

**Affiliations:** The Medical School, University of Central Lancashire, Preston, Lancashire UK

**Keywords:** Research ethics, Research integrity, Peer review, Epidemiology, Modelling, Bioethics, Principlism

## Abstract

Should research projects involving epidemiological modelling be subject to ethical scrutiny and peer review prior to publication? Mathematical modelling had considerable impacts during the COVID-19 pandemic, leading to social distancing and lockdowns. Imperial College conducted research leading to the website publication of a paper, Report 9, on non-pharmaceutical interventions (NPIs) and COVID-19 mortality demand dated 16th March 2020, arguing for a Government policy of non-pharmaceutical interventions (e.g. lockdowns, social distancing, mask wearing, working from home, furlough, school closures, reduced family interaction etc.) to counter COVID 19. Enquiries and Freedom of Information requests to the institution indicate that there was no formal ethical committee review of this specific research, nor was there any peer review prior to their online publication of Report 9. This paper considers the duties placed upon researchers, institutions and research funders under the UK ‘Concordat to Support Research Integrity’ (CSRI), across various bioethical domains, and whether ethical committee scrutiny should be required for this research.

## Introduction

This commentary considers the question, ‘Should research projects involving epidemiological modelling be subject to ethical scrutiny and review?’ Mathematical modelling has had considerable global impacts on human life during the COVID-19 pandemic, and modelling research has been used to argue for population-level health interventions such as social distancing and lockdowns [1]. Imperial College conducted research leading to the website publication of a paper, Report 9—Impact of non-pharmaceutical interventions (NPIs) to reduce COVID-19 mortality and healthcare demand dated 16^th^ March 2020 [1]. The paper argued for a Government policy of non-pharmaceutical interventions (e.g. lockdowns, social distancing, mask wearing, working from home, furlough, school closures, reduced family interaction etc.) intended to counter infection with COVID 19. UK Government spending implementing these and other interventions escalated to £410 billion [2]. For comparison, the 2020/21 budget for NHS England was £129 billion [3]. Report 9 was widely publicised by the first author, central to the implementation of UK COVID 19 health interventions and in turn, informed models for global interventions [4].

From an ethical perspective, presumably the NPIs advocated by the authors on the basis of the modelling research were presumably intended to have a beneficent purpose, but had other unintended, although foreseeable, consequences extending across various bioethical domains. The traditional four bioethical domains include autonomy, beneficence, non-maleficence and justice [5]. We consider whether all four domains were affected by the modelling research, its publication and the policies it advocated.

NPIs such as lockdowns curtailed personal freedom to associate with others and conduct family life and compulsion to wear a face mask affected individual autonomy. Modelling predicted many deaths, affecting people’s anxiety and mood levels, reducing the ability to obtain medical help, or increasing levels of substance misuse and so consideration of the domain of non-malificence is relevant. The disbursal of large sums of money to implement the lockdowns with borrowing requirements to fund furlough and alleviate any poverty caused meant that the domain of social justice was also relevant to consideration.

Although it could be argued that modelling did not require ethical committee discussion as no live subjects were involved in the research methods itself, such modelling is deemed research and UK universities are signatories to ‘The Concordat to Support Research Integrity’(CSRI) published in 2012 and 2019 [6, 7]. Both versions of the CSRI predate the COVID-19 modelling research**.** The CSRI upholds the Singapore Statement on Research Integrity [8] and applies ‘to all fields of research’ and ‘all disciplines in which research is undertaken’, research itself being defined in the CSRI as a *‘a process of investigation leading to new insights, effectively shared… It includes work of direct relevance to the needs of commerce, industry, and to the public and voluntary sectors; scholarship; the invention and generation of ideas, images, performances, artefacts including design, where these lead to new or substantially improved insights; and the use of existing knowledge in experimental development to produce new or substantially improved materials, devices, products and processes, including design and construction’.*

## Main text

The CSRI (2019) noted that *standards* of research integrity should include ‘care and respect for all participants in research, and for the subjects, users and beneficiaries of research, including humans, animals, the environment and cultural objects’. This implies a duty of care and respect not only for the subjects involved in any research process but also for the intended ‘beneficiaries of research’. This expands the focus of ethical consideration beyond just the participants in the research process. The people potentially affected by the research – ‘beneficiaries’—also require consideration [7]. This is vital as it can be interpreted that researchers should consider the future effects of the research on a far wider community than just the ‘subjects’ directly involved within the research.

Furthermore the CSRI (2019) requires that researchers themselves ‘ensure that all their research is subject to active and appropriate consideration of ethical issues’ [7]. This *active* consideration would, presumably, go beyond any passive presumption of a personal prerogative to engage in any research the researcher wishes to conduct and instead proactively seek ethical consideration of their research, and particularly where bioethical principles are at stake, and multiple ‘beneficiaries’ and stakeholders potentially affected by the proposed research.

The earlier document, The Singapore Statement on Research Integrity (2010), stated that: ‘Researchers and research institutions should recognise that they have an ethical obligation to weigh societal benefits against risks inherent in their work’ [8].

Report 9 describes the COVID 19 pandemic as a “major global health threat” and makes the substantial claim that COVID 19 is a “virus with a comparable lethality to H1N1 influenza in 1918” [1]. The lethality of the 1918 pandemic was such that the “number of deaths was estimated to be at least 50 million worldwide with about 675,000 occurring in the United States” [9]. The equivalence to the 1918 pandemic is repeated and emphasised through Report 9. “The last time the world responded to a global emerging disease epidemic of the scale of the current COVID-19 pandemic with no access to vaccines was the 1918–19 H1N1 influenza pandemic” [1].

The intention of the research to inform and affect policymaking is explicitly noted – “Here we present the results of epidemiological modelling which has informed policymaking in the UK and other countries in recent weeks.” Only two strategies are presented as options – mitigation and suppression, with “suppression as the preferred policy option”. The authors were aware of the potential societal impact of their recommendations and that these interventions were intended to be extensive and last for many months. They concluded that such suppression “will minimally require a combination of social distancing of the entire population, home isolation of cases and household quarantine of their family members. This may need to be supplemented by school and university closures, though it should be recognised that such closures may have negative impacts on health systems due to increased absenteeism. The major challenge of suppression is that this type of intensive intervention package – or something equivalently effective at reducing transmission – will need to be maintained until a vaccine becomes available (potentially 18 months or more).” The authors were therefore aware of the very grave consequences that might follow from their research admitting there might be “social and economic costs of the interventions”. The researchers actually acknowledge an absence of ethical consideration in the paper, saying “we do not consider the ethical or economic implications” of either strategy [1].

In the methods section the authors note their modelling uses values derived from numerous layered assumptions, e.g. “We assumed an incubation period of 5.1 days”, “we make a baseline assumption that R0 = 2.4”, “We assume that symptomatic individuals are 50% more infectious than asymptomatic individuals.”, “Individual infectiousness is assumed to be variable.”, “On recovery from infection, individuals are assumed to be immune to re-infection in the short term.”, “Infection was assumed to be seeded in each country at an exponentially growing rate (with a doubling time of 5 days)”, “We calculate bed demand numbers assuming a total duration of stay in hospital of 8 days if critical care is not required and 16 days (with 10 days in ICU) if critical care is required.”

Some assumptions are based on personal communication, rather than peer reviewed publication e.g. assumptions about re-infection “Evidence from the Flu Watch cohort study suggests that re-infection with the same strain of seasonal circulating coronavirus is highly unlikely in the same or following season (Prof Andrew Hayward, personal communication).”

Other assumed figures are “adjusted’ from other published work e.g. “The IFR estimates from Verity et al. have been adjusted to account for a non-uniform attack rate giving an overall IFR of 0.9% (95% credible interval 0.4%-1.4%) [10]. Hospitalisation estimates from Verity et al. were also adjusted in this way and scaled to match expected rates in the oldest age-group (80 + years) in a GB/US context.” The reference in Report 9 is to a preprint, (published March 13^th^ 2020) that is a paper before peer review, and explicitly labelled thus:

This article is a preprint and has not been peer-reviewed. It reports new medical research that has yet to be evaluated and so should not be used to guide clinical practice [10].

medRxiv counsels, regarding preprints, “We also urge journalists and other individuals who report on medical research to the general public to consider this when discussing work that appears on medRxiv preprints and emphasize it has yet to be evaluated by the medical community and the information presented may be erroneous” [10].

The Verity et al. paper disclosed similar financial support to Ferguson et al.’s paper. Ferguson helped conceive the Verity et al. study, had input in the analysis, and is listed as a co-author. Report 9 therefore referenced its assumptions with a pre-print of another paper by the same team. The Verity preprint extrapolated its conclusions from data including 2,946 deaths in China, six flights from China, and a cruise ship to make estimates of the case fatality rate [10]. The Verity et al. paper was later published in The Lancet (Infectious Diseases) on March 30^th^ 2020.

The precise nature of the modelling algorithm is not published within Report 9 [1].

The results section includes a prediction that “in an unmitigated epidemic, we would predict approximately 510,000 deaths in GB and 2.2 million in the US”. With mitigation “we predict there would still be in the order of 250,000 deaths in GB, and 1.1–1.2 million in the US” [1].

Predictions of deaths of this scale were simultaneously reiterated in interviews given by Ferguson to the press e.g. reported in the Daily Telegraph in March 17^th^ 2020, the day after Report 9 was published [11]. The event was described as “a jaw-dropping press briefing” and the data “terrifying”.

### Previous modelling work

Imperial College’s research modelling infectious diseases advocated the mass culling of sheep and cattle during the 2001 outbreak of foot-and-mouth disease [12]. Ferguson’s paper concluded: “Hastening the slaughter of animals with suspected infection is predicted to slow the epidemic, but more drastic action, such as “ring” culling or vaccination around infection foci, is necessary for more rapid control. Culling is predicted to be more effective than vaccination.” [12]. Some six million sheep and cattle were slaughtered [13].

In 2002, Ferguson et al. predicted that up to 150,000 people could die from exposure to BSE (Bovine Spongiform Encephalopathy) in beef and lamb, in a worst-case scenario, stating “we estimate the 95% confidence interval for future vCJD mortality to be 50 to 50,000 human deaths considering exposure to bovine BSE alone, with the upper bound increasing to 150,000 once we include exposure from the worst-case ovine BSE scenario examined.” [14]. There were eventually only 177 recorded human deaths from BSE [15].

In 2005, Ferguson et al. published a paper on ‘an emerging influenza pandemic’ in Southeast Asia [16] concluding that action was required to “prevent millions of deaths” and that “the costs of failure are potentially so catastrophic that it is imperative for the international community to prepare now, to ensure that containment is given the best possible chance of success.” In media interviews Ferguson predicted avian flu could kill up to 200 million people e.g. in The Guardian, Ferguson was quoted thus: "Around 40 million people died in 1918 Spanish flu outbreak. There are six times more people on the planet now so you could scale it up to around 200 million people probably." [17]. In an earlier interview Ferguson advocated urgent action – “Prof Ferguson warned that failure to take action swiftly enough would result in catastrophe.” and “that an international stockpile of 3 m courses of antiviral treatment would be enough to contain an outbreak.” [18]. By the end of 2005, there had been 74 avian flu deaths worldwide [19].

In 2009, a government estimate provided by SAGE, based on Ferguson’s work, was that in a “reasonable worst-case scenario” swine flu would cause 65,000 British deaths [20]. In an interview with The Guardian Professor Ferguson predicted that swine flu would affect a third of the world’s population and that the UK would have a “flu season which is perhaps three times worse than usual” and that it was “possible it could be like 1957 – where about three to four out of 1,000 people who were infected died and overall about 3 million to 4 million people died that year because of the pandemic.” [21]. Later in the year Ferguson advocated the closure of schools in the UK [22], based on a paper he co-authored [23]. In 2009 the UK stockpiled enough of the antiviral drug, Tamiflu, to treat 80% of the population and spent £136 million on the antiviral drug Relenza [24]. The U.K. Government eventually recorded 457 deaths from swine flu [20].

The authors of the paper emphasise their belief that COVID-19 shares the lethality of the 1918 H1N1 influenza virus, describing it as a “virus with a comparable lethality to H1N1 influenza in 1918” [1]. The latter virus is estimated to have caused 50 million deaths (CDC, 2021) in 1918 when the global population was only 1.8 billion [9].

The summary, introduction, methods, results and discussion sections of Report 9 all refer to non-pharmaceutical interventions to mitigate deaths. There are 25 mentions of the word ‘policy’ and two mentions of ‘policymaking’. There are no mentions of the words ‘hypothesis’, ‘ethics’, ‘incorrect’ or ‘wrong’. The word ‘ethical’ only appears in the sentence “We do not consider the ethical or economic implications of either strategy here…”.

The imposition of policies advocated by the authors of Report 9 led to social distancing measures in the UK, with repeated closures of hospitality business, other businesses, universities, schools. A furlough scheme was required to support earnings, with consequent costs to the Exchequer including additional schemes such as Track and Trace to support social distancing, of borrowing of £410 billion [2].

NHS primary and secondary health care was partially closed to routine surgical procedures and face to face consultations with medical staff. Over 12 million patients had necessary NHS treatment delayed [25]. There was a rise of around 40,000 non-COVID deaths at home in England and Wales in 2020 -21 [26]. An example of a consequence of globally adopted NPIs includes a reduction in cancer screening opportunities with a loss of 300,00 mammograms in Ontario with 4,119 undiagnosed cancers in Quebec during the first wave of COVID-19 [27].

Over the course of a year of lockdowns, the Royal College of Psychiatrists estimated a rise of 10 million cases of anxiety and depression with 1.5 million of these being children and adolescents [28]. There was a rise in the incidence of deliberate self harm [29], and a 20% rise in alcohol related in deaths in 2020 in England and Wales [30].

## Conclusion

One viewpoint would be that a narrow interpretation of research ethics is appropriate for modelling of diseases – that a research methodology which does not directly involve humans dictates that ethical consideration is not required, and that an insistence upon ethical review would be an impediment to researchers’ freedom, the speed of research necessary for managing a pandemic and hold back an academic institution’s ability to compete and earn vital revenue.

Urgency in modelling a pandemic could be understood during an emergency situation, but this does not mean that peer review should be skipped before publication nor when stringent social policies are being recommended should ethical review be omitted. A rapid review mechanism by a standing ethical committee could be enabled for case-by-case ethical evaluations in context, especially when modellers make recommendations for sweeping policy interventions affecting every facet of society, particularly if no risk/benefit assessment is made in the article making those very recommendations.

Imperial College modelling research in Report 9 met the definition of research contained within the CSRI [7]. Report 9, and the research underpinning it, was not subject to the scrutiny of an ethics committee nor subjected to an independent peer review process before publication and was therefore injected directly into the heart of Government and used for policy making without the safeguards of ethical review or peer review.

Report 9 was explicitly to affect health and social policies, advocating for stringent non-pharmaceutical interventions. The authors could not argue they were unaware of the profound societal impact of the research on what the CSRI would optimistically term ‘beneficiaries’ of that research. The authors acknowledge the policy they advocate could be associated with ‘enormous social and economic costs’ with ‘significant impact on health and well-being’.

The CSRI of 2012 [6], and 2019 [7] required researchers to ‘ensure that all their research is subject to active and appropriate consideration of ethical issues’ [7].

The Report and associated press briefings directly compared the lethality of the COVID19 and 1918 influenza pandemics [1]. Reporters described the briefings as ‘terrifying’ [11]. The mortality claims might have been challenged in independent peer review and explored in an ethics review which might also have considered the potential effects of such research involving bioethical domains. Past modelling predictions of infectious deaths on which policies had been advocated were excessively high [12, 14, 17, 18, 20, 21]. The ethical review might have considered the track record of past modelling research, past effects on the economy and the need to include explicit warnings that projections could be wrong, and thus the risks of implementing advocated policy. None of the 2020 predictions for COVID death figures in Report 9 were met. There was no consideration that enacted policy, based on erroneous projections, might directly or indirectly be followed by adverse events; for instance psychological effects such as the near doubling of the prevalence of anxiety and depression in children [31] or increased deaths due to non-treatment of cancer and coronary disease [32], reduced life expectancy and increased mortality due to economic shock [33], and millions of patients awaiting operations in the NHS [25] amongst others.

External funding by third parties with remits in health and social policy may have made ethical review prudent [34–36].

Ethical review should not be viewed as an unnecessary impediment to research. Obtaining an ethical review would not have been detrimental to the research process, rather it might have enriched and benefitted the eventual Report, by incorporating additional viewpoints.

Report 9 spoke directly to the heart of Government seeking to effect change in health policies and intervene in health-related matters and healthcare, arguing to curtail human freedoms via social distancing; logically affecting people’s freedom to earn, to go to school or University, and affecting their ability to associate with friends and family. Such epidemiological modelling, intended to affect health and social policy as here, with widespread societal consequences should warrant ethical discussion at the outset [37–39].

### Suggested actions

• Research—regardless of methodology—with potential consequences for humans, animals, the economy or the environment—should be subjected to ethical committee review.
• Embed the teaching of research and professional ethics into all UK University courses, particularly science courses.
• Some professional regulatory body for University employed researchers, akin to the GMC, to inquire into their ethical behaviours and ability to regulate accordingly.
• Consideration of a professional oath or code in science -based University courses similar to the Hippocratic Oath used in medical courses [40].
• Requiring all University researchers to undertake refresher courses in research and other professional ethics.
• Government should consider the ethical dimension to research it considers, and in important policy decisions, only relying on research that is actively ethically approved, peer reviewed, scientifically replicated and after a full risk / benefit analysis.
• Universities and funders of research should ensure ethical and peer reviews of research are carried out on research they are directly or indirectly affiliated with.
• Journals should only publish original research papers where researchers can demonstrate a prospectively acquired ethical review.
• Research should only be published or promoted after ethical and independent peer review.

## Data Availability

Not applicable.

## Abbreviations

CSRI: Concordat to Support Research Integrity
NPI: Non-Pharmaceutical Intervention
